# Malaria in Kenya's Western Highlands

**DOI:** 10.3201/eid1109.041131

**Published:** 2005-09

**Authors:** G. Dennis Shanks, Simon I. Hay, Judy A. Omumbo, Robert W. Snow

**Affiliations:** * US Army Medical Research Unit–Kenya, Nairobi, Kenya;; † University of Oxford, Oxford, United Kingdom;; ‡ Kenya Medical Research Institute, Nairobi, Kenya;; § John Radcliffe Hospital, Oxford, United Kingdom

**Keywords:** Plasmodium falciparum, malaria, epidemiology, highland, drug resistance, Kenya., historical review

## Abstract

Records from tea estates in the Kericho district in Kenya show that malaria reemerged in the 1980s. Renewed epidemic activity coincided with the emergence of chloroquine-resistant *Plasmodium falciparum* malaria and may have been triggered by the failure of antimalarial drugs. Meteorologic changes, population movements, degradation of health services, and changes in *Anopheles* vector populations are possible contributing factors. The highland malaria epidemics of the 1940s were stopped largely by sporontocidal drugs, and combination chemotherapy has recently limited transmission. Antimalarial drugs can limit the pool of gametocytes available to infect mosquitoes during the brief transmission season.

## Abstract 2

Reemergence of epidemics in tea plantations will likely result in antimalarial-drug resistance.

Epidemic malaria is a term applied to describe *Plasmodium falciparum* transmission characteristics of the highlands of East Africa and the Horn of Africa. These areas are fringe regions between stable and unstable transmission, which are affected to some degree by annual variations in rainfall but primarily by low ambient temperature. Such areas are often densely populated and of economic and political importance because of their agricultural potential. Much attention has been given to the increasing frequency and clinical costs associated with epidemics among highland populations in Africa (*1**–**3*). We examine historical and contemporary data to define the long-term epidemiologic transition of malaria in 1 district of the western highlands of Kenya. We use these data in support of our hypothesis that drug resistance is a key element in highland malaria epidemics in East Africa, and we examine how past control might guide future efforts to reduce the clinical impact of epidemics.

## Geography and Land Use

**Figure 1:**
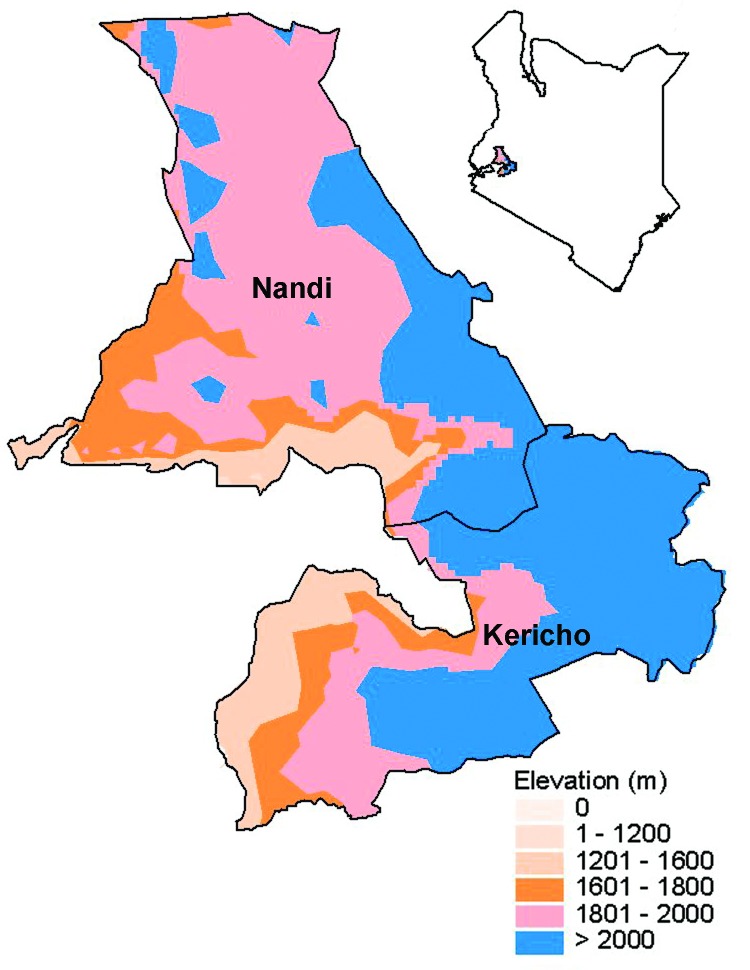
Map of Kenya showing the Nandi and Kericho districts.

**Figure 2:**
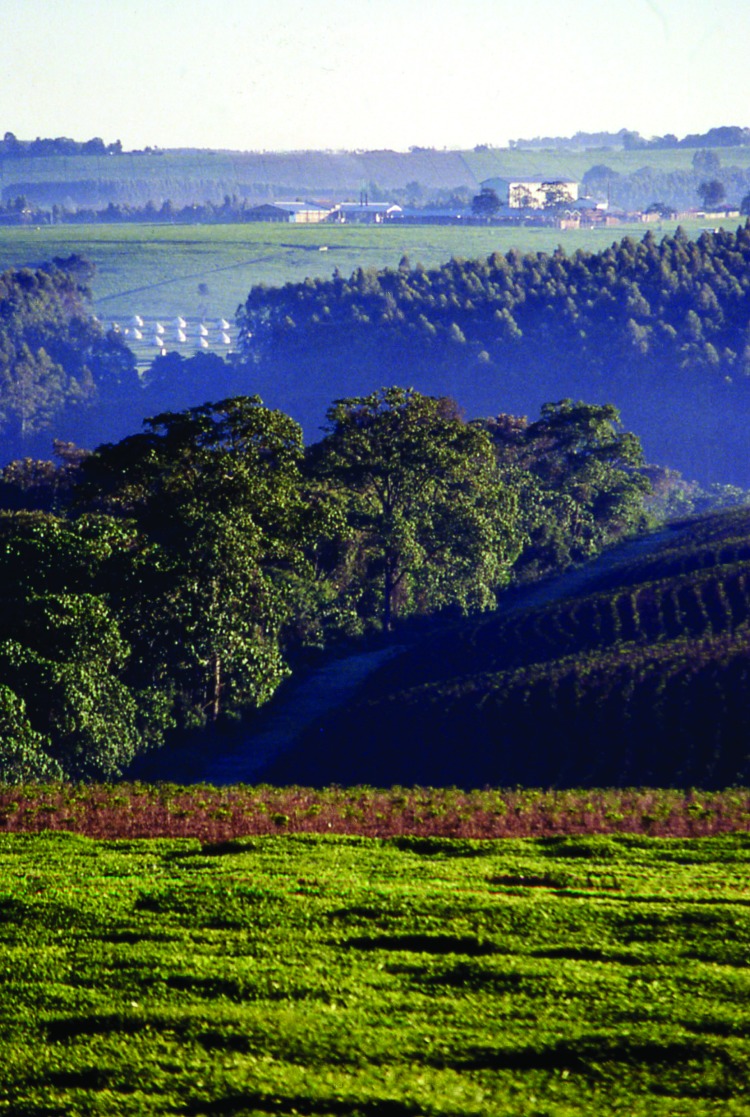
Kericho, Kenya, tea plantation in 1998.

The Kericho district is located on the western side of the Great Rift Valley in a highland area near Lake Victoria (elevation 1,600–3,000 m above sea level) (Figure 1). The soils are deep and well drained. Because of adequate and reliable rainfall, the district can produce a surplus of crops (forestry and horticultural products, pyrethrum, cereals, fruit trees, tea). Tea is grown on self-contained farms, called estates, that usually have ≈1,000 workers tending <1 square mile of tea bushes. Two tea plantations (Figure 2), each consisting of 18 estates, employed 18,000–18,500 workers in 1998. Employees reside at the estate with 3 to 4 dependents each (*4*).

## Migration and Mobility

Arrival of the railroad in the early 20th century and improvement of roads facilitated movement from the Kenyan coast and lake districts to the highlands, which were thought free of malaria. European settlement began before World War I as an extension of other farming areas in the Rift Valley. A large proportion of the tea estate labor force were recruited from the Lake Victoria region (elevation <1,000 m) or from Kisii (elevation 1,500 m) in Nyanza province. The Lake Victoria region is an area of holoendemic malaria whose inhabitants often have asymptomatic infections (*5*). Tea pickers are given 1 month leave per year to return to their families in their natal village. Over time, dependents of the tea pickers have come to live on the estates. Travel back and forth from the highland estates to the Lake Victoria lowlands is common (*4*).

## Climate

Variations in climate affect the distribution and abundance of malaria vectors. The effects of temperature on the transmission cycle are manifold, but its specific effect on sporogonic duration and mosquito survival is critical. When the temperature is <18°C, transmission is unlikely because few adult mosquitoes (0.28%) survive the 56 days required for sporogony at that temperature and mosquito abundance is limited by long larval duration. At 22°C, sporogony is completed in <3 weeks, and mosquito survival is sufficiently high (15%) for the transmission cycle to be completed. The potential number of infective mosquitoes reaches a peak at 30.6°C, after which it decreases rapidly. The relationship between mosquito abundance and rainfall is complex and best studied when temperature is not limiting. *Anopheles gambiae* s.l. breed more prolifically in temporary and turbid water bodies, while in permanent bodies predation is an important limitation. Rainfall is a good indicator of vectors, their survival, and the potential for malaria transmission. Long-term, complete meteorologic data are available from the Tea Research Foundation. Analysis of the meteorologic relationships of malaria in Kericho has been reported (*6**–**8*).

## Health Service Provision and Drug Resistance

The public health sector in sub-Saharan Africa has been overwhelmed by AIDS, lack of money, and other problems. In Kenya, population increases of 3% to 4% per year, an inflationary economy in the face of fixed health service wages, and administrative failures have depleted public health services (*9*). However, this situation does not apply to the tea plantations, where private agricultural companies maintain a healthy, productive workforce (*3*). Whatever the cause of recent increases in malaria in the western highlands of Kenya, the tea plantation health systems have continued identifying, counting, and treating malaria infections among their workers. Since 2000, worker residences have been sprayed with insecticide every 4 months, primarily for pest control. This measure is unlikely to have differentially influenced the malaria data because both plantations were using the same regimen.

Chloroquine was used to treat uncomplicated malaria infections in Kenya from the 1950s until the national drug policy was changed in 1998 (*10*). Chloroquine resistance in Kenya was first documented in 1979 in a tourist who had visited Kenya. After resistance became widespread (*11*), chloroquine continued to be used as the first-line antimalarial drug for another 19 years. A 1985 survey indicated that parasites from the adjacent Nandi district were still sensitive to chloroquine (*12*), whereas by 1996 chloroquine was unable to clear 50% of clinical infections in children by day 7 (*13*).

## Sources of Clinical Data since 1900

We used 3 principal sources of data. The first source was reports in the Kenya national archives. The second was local and international journals reports of malaria epidemics in the region. The third was hospital data from 2 adjacent tea plantations directly accessed to obtain the temporal and secular patterns of malaria in Kericho. Inpatient data have been located in admission registers of the hospital at tea plantation 1 from 1965 to 2004 (*3**,**14*). The second tea plantation hospital is adjacent to plantation 1 and since 1970 has maintained a weekly infectious disease notification system that identifies all blood smear–positive malaria cases. Although the system includes inpatients, it consists mostly of outpatients. Although the 2 hospitals are adjacent, the populations served are separated according to the company of employment. Combined, these reports, data, and anecdotes build a qualitative and quantitative picture of Kericho epidemics during the 20th century.

## Epidemics

### 1918–1919 and 1928

During the 19th century, local malaria transmission was nonexistent or negligible (*15*). Increasing trade and transport led to major population movements from 1906 onwards. Movement of people associated with the opening of civil and military posts probably introduced malaria into the highlands. During World War I, soldiers from Kericho were recruited to fight against the German forces in Tanganyika. With troop demobilization and resettlement in 1918 and 1919, a malaria epidemic followed the influenza pandemic (*15*). Further development in this region, including the completion of the Ugandan railway from the Mau escarpment to the malaria-endemic Lake Victoria region, increased movement of people and parasites.

In 1928, an epidemic in Kericho district that involved 1,727 hospital case-patients occurred (*15*). Epidemics were also reported in 1931, 1932, 1934, 1937, and 1940 (*5*). Malaria was much more severe in highland workers than in their presumably functionally immune counterparts from Lake Victoria (*16*). Health authorities used mass drug administration for epidemic control, dispensing 57,600 ten-grain (600 mg) doses of quinine in 1 month (*16*).

### 1939–1948 and Introduction of Control

Epidemic malaria became a major infection on the tea estates during World War II (*17*) because soldiers returning from Ethiopia through the malarious coastal areas were encamped along the adjacent railway (*18*). Epidemics appeared to be caused by the large pool of parasitemic soldiers who infected the local mosquitoes during the brief mid-year period suitable for malaria transmission at high altitudes and occurred mostly within the military camp, the township of Kericho, and the tea estates, while sparing most "native reserve areas" (*18*). Removal of the military camp at the end of the war did not stop the now indigenous malaria transmission (*17*).

**Figure 3:**
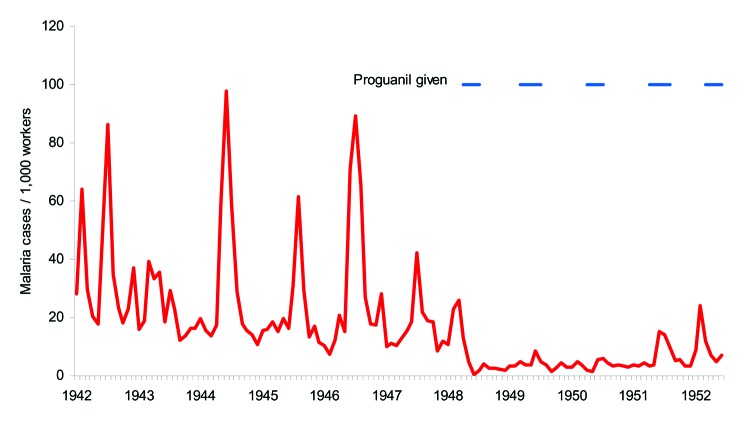
Monthly malaria cases on the Kericho tea estates, Kenya, 1942–1952, showing mass administration of proguanil. Data were obtained from Strangeways-Dixon (*17*) and hospital records.

Given apparently fragile malaria transmission in the agriculturally important western Kenyan highlands, interventions in Kenya were often first tried in Kericho. Proguanil was a safe chemoprophylactic agent that had just been developed as the result of an emergency wartime project of the British and Australian armies. The medical officer for plantation 1, D. Strangeways-Dixon, used mass drug administration of proguanil, as the British army had been doing in war areas (Figure 3) (*17*). In March 1948 (before the epidemic in the rest of the district), prophylactic proguanil was given twice a week (a single 100-mg tablet) to all plantation 1 employees and their dependents. This strategy decreased malaria incidence in June and July 1948 (0.5 and 2.0 cases per 1,000 population, respectively, compared with nearly 10 times that number the previous year). Two rounds of house spraying with DDT in 1949 (March and June) complemented the prophylactic measures. During this period, malaria was virtually controlled. Proguanil did not eliminate epidemics, however, and in 1952 the prolonged rainy season shifted the epidemic period from July to January, and malaria returned before the start of proguanil prophylaxis. Reinstitution of proguanil resulted in epidemic control, probably by blocking further transmission.

### Intermission, 1960–1980

Malaria in the Kericho district was largely imported by persons traveling back from Lake Victoria. For the years in which data were provided in the medical officer's reports (1966, 1967, 1970, 1975, and 1976), 95 deaths occurred among 5,686 malaria patients admitted to Kericho district hospital (case-fatality rate 1.7%). Recent studies on the Kenyan coast showed an overall case-fatality rate of 3.5%, varying greatly depending on the clinical syndrome that caused hospitalization (*19*). At the Kericho tea estates, malaria ceased to be a major problem from 1960 to 1980, which roughly coincides with the period during which chloroquine was fully effective against *P. falciparum* malaria.

### Reemergence of Seasonal Epidemics

**Figure 4:**
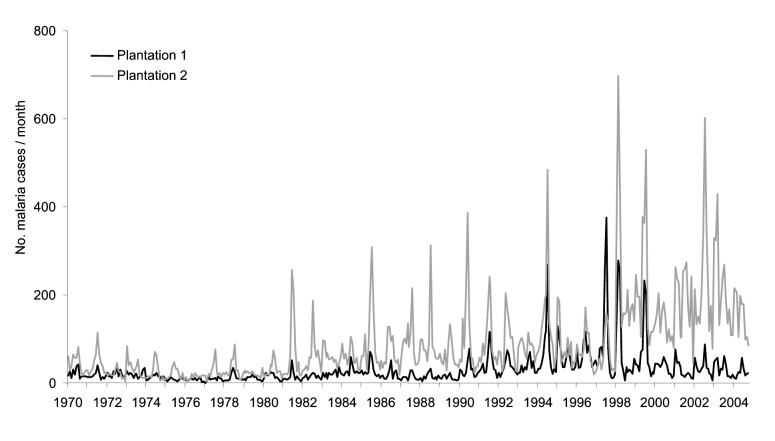
Monthly malaria incidence at 2 adjacent tea plantations in Kericho, Kenya, 1970–2004. Plantation 1 data are from inpatient admission registers, and plantation 2 data are from weekly malaria slide reports that include outpatients.

**Figure 5:**
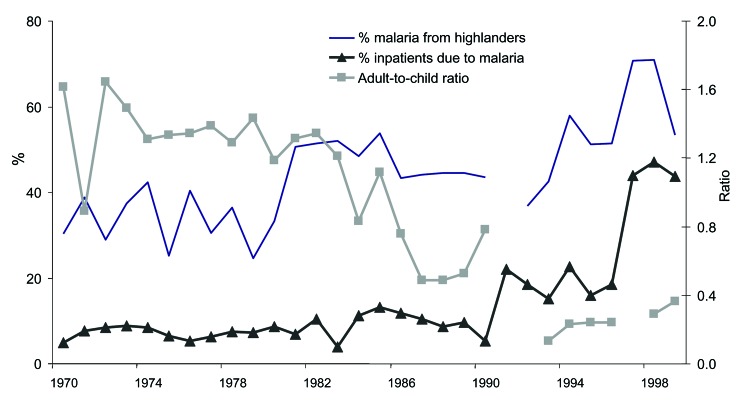
Annual malaria inpatient characteristics from tea plantation 1 in Kericho, Kenya, 1970–1999. Percentage of malaria patients compared with percentage of all hospital admissions, percentage of malaria inpatients of highland (>1,500 m) family origin, and ratio of adults to children (<15 years of age) of all malaria inpatients are shown annually as collected from the same admission registers. Gaps indicate missing data.

In Kericho, annual mid-year malaria epidemics began in 1990 at plantation 1, although epidemic peaks were evident in 1981 at plantation 2 (Figure 4). Increasing malaria incidence was not related to overall warmer temperatures but still depended on the annual pattern seen in the 1940s in which malaria would increase after the rains in March through April and decrease after the onset of cool weather in July (*8**,**9*). Malaria admissions at plantation 1 increased accordingly, from 5% of all admissions in 1970 to 47% in 1998 (Figure 5). However, before this increase, other changes were noted such as increase in the percentage of malaria inpatients of highland (>1,500 m) origin in 1981 and decrease in the adult-to-child ratio of inpatients in the mid-1980s (Figure 5) (*20*). A high adult-to-child ratio of malaria indicates a less immune population in which adults become symptomatic when infected with malaria, which is unusual in areas of high transmission (*20*). The case-fatality rate for malaria admissions at plantation 1 from 1965 to 1972 was 1.3%. The rate increased to 6% from 1990 to 1998, despite good medical and nursing inpatient care (*14*). On the Kericho tea estates, most malaria deaths are the result of severe anemia in young children, not cerebral malaria (*21*). In other areas of Kenya, chloroquine resistance increased case-fatality rates, and this trend could be reversed by using more effective antimalarial drugs (*22*).

**Figure 6:**
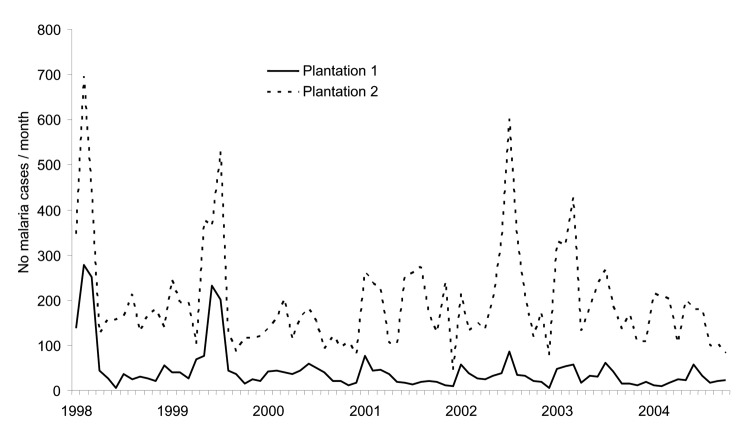
onthly malaria incidence at 2 adjacent tea plantations in Kericho, Kenya, 1998–2004. Shown are the same data in Figure 4 in an expanded scale. See section on sources of clinical data since 1900 to distinguish outpatient and inpatient composition.

**Figure 7:**
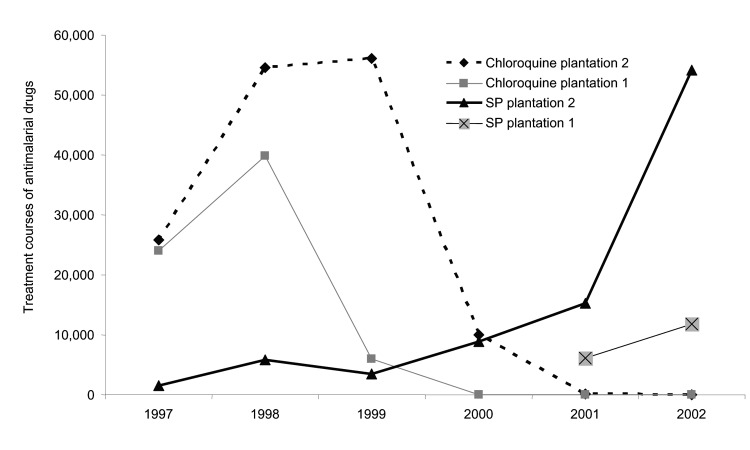
Annual antimalarial drug purchases recorded in the respective tea plantation hospital pharmacy records, Kericho, Kenya, 1997–2002, showing the discontinuation of chloroquine as sulfadoxine-pyrimethamine (SP) became first-line therapy. Records of SP purchases at plantation 1 prior to 2001 were not available.

Malaria incidence on the 2 adjacent tea plantations generally followed each other closely, despite separate worker populations and medical systems. A striking divergence, however, was noted in 2002, when heavy rains followed 2 years of low malaria incidence at both plantations because of drought (Figure 6) (*23*). This difference was confirmed when malaria incidence between outpatient malaria patient visits at plantation 1 (*4*) and outpatient visits at the Kericho district hospital were compared (*24*). Absence of increased malaria at plantation 1 in 2002, while epidemic conditions existed in both adjacent plantation 2 and the surrounding district, is difficult to explain on the basis of weather, human migration, medical access, or vector population changes (*24*). One change that coincided with the abatement of malaria at plantation 1 was a switch in first-line malaria treatment for outpatients from chloroquine to sulfadoxine-pyrimethamine (SP) in 1999 (Figure 7). A prospective malaria surveillance project involving febrile outpatients on plantation 1 showed that after the substitution of SP for chloroquine, *P. falciparum* gametocyte rates decreased in young children from 5% (1998) to <1% (2002), while remaining unchanged in adults (<1%) (*4*). This result occurred in the absence of any change in asexual parasitemia (≈50% of febrile pediatric outpatients were positive) over the same period. Although no simultaneous surveillance of malaria parasites occurred at plantation 2, which switched to SP in mid-2000, the appearance of epidemic malaria in the 1980s, as well as continued seasonal malaria on plantation 2 after 2000, suggests that other factors contributed to local transmission.

At least 3 chemotherapeutic explanations exist for progressive decrease in gametocytes on plantation 1 without a significant effect on the percentage of febrile children with asexual parasites. 1) Chloroquine-resistant *P. falciparum* strains produce more gametocytes and infect more mosquitoes than chloroquine-sensitive strains, which may explain why chloroquine resistance has spread rapidly across Africa (*25*). The removal of chloroquine drug pressure on plantation 1 in 1999 likely reduced the number of infected mosquitoes. 2) Combination chemotherapy with pyrimethamine increased from <20% of all treatment courses on plantation 1 in 1998 to 85% of all treatment courses by 2001. Pyrimethamine blocks the development of parasites in the mosquito (*26*). Even increased numbers of gametocytes produced after SP treatment are transmitted poorly through the mosquito because of the antifolate action of pyrimethamine (*27**,**28*). The sporontocidal effect of the pyrimethamine component of SP is separate from its ability to clear parasites from the blood. Pyrimethamine had been used successfully as part of mass drug administration to block transmission in the adjacent Nandi district during the 1950s (*29*). 3) The use of artemisinin-containing combinations increased on plantation 1 from 3% of all treatment courses in 2001 to 9% in 2002, whereas artemisinin combinations represented <2% of all antimalarial drugs purchased at plantation 2 in 2002. From 2000 to 2002, 52% of hospitalized malaria patients at plantation 1 received an artemisinin compound compared with 36% at plantation 2, but this percentage represented a relatively small number of total malaria cases. Artemisinin compounds killed gametocytes and blocked transmission in Thailand (*30*) and The Gambia (*31*). Increased use of artemisinin combinations primarily for sick children on plantation 1 would have limited the pool of gametocytes available to infect mosquitoes. The combined effect of discontinued chloroquine and increased use of SP and artemisinin combinations resulted in too few infective mosquitoes to start an epidemic during the brief highland transmission season.

## Combining the Evidence

Reemergence of mid-year malaria epidemics in the western Kenyan highlands has progressed to annual incident peaks, which suggests that this area is now one of seasonal rather than epidemic malaria (*9**,**24*). Reemergence is the proper term because mid-year malaria increases were common during World War II, and Kericho has not been free of malaria for over 60 years (Figure 3). What has changed to cause malaria to revert to its earlier pattern after a long quiescence? Possible factors are changes in climate variability, population movements, decrease in quality of health services, changes in mosquito vectors, and antimalarial drug resistance.

Doubts exist as to the plausibility of climate change as proximate cause of epidemic malaria because global warming cannot explain the World War II epidemics. Dramatic increases in malaria during the 1990s are not mirrored by prospectively collected climate data from Kericho (*7*). No warming trend or increase in temperature records that extend back nearly a century was observed at several points in East Africa (*8*). Extensive comparison of temperature, rainfall, and malaria records in Kericho after 1965 has not indicated any convincing multiple-year link between either rainfall or temperature and malaria (*32*). Furthermore, continent-wide trends in malaria transmission suitability during the 20th century do not show the Kenyan highlands as an area of substantial change (*33**,**34*).

Malaria epidemics during World War II were probably the result of population movement; stationing of soldiers in Kericho after military operations in malarious areas started the epidemic. This epidemic was presumably the result of the focal concentration of imported human gametocyte carriers who infected mosquitoes during the brief mid-year period when local transmission was possible. Extensive travel between holoendemic Lake Victoria and Kericho has occurred at least since World War II. Prospective data suggest that persons returning from the malaria-endemic lowlands transport malaria parasites up the mountain to Kericho as asymptomatic or symptomatic infections (*4*). Population movements alone cannot explain the lack of substantial malaria from 1960 to 1980. Although population movements are important in introducing malaria into a malaria-free area, the highlands of western Kenya have not been free of malaria infections since World War I.

In the last 25 years, Africa has seen a decrease in the quality and quantity of public health services as result of rapidly expanding population, decrease in purchasing power of African currencies, severe institutional or organizational problems, and increase in HIV infection. Yet Kericho tea plantations have maintained high-quality health services to a defined population (*3*). Whatever the problems of the African district hospital, the interest of the tea companies in maintaining healthy workers eliminates decrease in healthcare services as an explanation of highland malaria epidemics.

Three entomologic surveys from 1946 to 1948 established that *An. gambiae* is the principal vector in Kericho with *An. funestus* playing a minor role (*35*). Investigations from 1998 to 2000 to characterize the mosquito vectors on the Kericho tea estates confirm the findings of Garnham obtained >50 years ago (*18*), which indicated that small numbers of *An. gambiae* in houses are malaria vectors (R. Dunton, pers. comm.). Unpublished studies conducted by Garnham on the same tea estates in the 1940s indicated that residual insecticide spraying of house walls could be very effective in controlling malaria. Modern vector control measures have not had any apparent differential effect on malaria in the 2 plantations, but their potential has not been fully explored. Similarly, long-lasting insecticide-impregnated bed nets have not been systematically implemented. Therefore, any major shift in species composition of the malaria vectors is unlikely to have contributed to malaria reemergence.

Drug resistance, specifically chloroquine resistance, may be key in the increase of highland malaria. The period without major malaria epidemics in the Kenyan highlands extends from chloroquine introduction until chloroquine resistance became widespread. Just as the parasites from nearby military camps caused the epidemics of World War II, the inability of chloroquine to eliminate parasites recreated a similar situation during the 1990s in the resident population. The increase in death rates of hospitalized malaria patients since 1990 also indicates chloroquine resistance and has been observed in other parts of Africa (*36*). Population increases that contribute to a decrease in access to health care can explain some highland malaria epidemiology, but this did not occur in Kericho, where number of workers and resident dependents has been stable (*20*). Drug resistance to chloroquine remains the leading explanation for the reemergence of highland malaria in the Kericho tea plantations and has been implicated in nearby areas (*37*). The absence of an epidemic on plantation 1 after use of more effective antimalarial drugs is additional evidence that the prime factor influencing highland malaria in Kericho is antimalarial drug resistance. Drug resistance is not a universal explanation for epidemic malaria in the highlands of East Africa, especially given the highly controlled nature of the tea estates, which is atypical of rural Africa. Kabale, Uganda (*38*), and Nandi, Kenya (*39*), are reminders that malaria is a focal disease susceptible to many factors that vary by specific location and season.

## The Future

Increasing chloroquine failure rates directly influence clinical outcomes and affect public health consequences in areas of marginal malaria transmission, such as Kericho. Small changes in the number of persons carrying infective gametocytes can initiate malaria epidemics through increased mosquito transmission. SP lowers transmission pressure by curing more infections and decreasing the infectivity of surviving gametocytes by the sporontocidal action of pyrimethamine (*26**–**28*). However, SP fails to cure uncomplicated malaria in East Africa and can only be viewed as an interim replacement for chloroquine (*40*). Appropriate case management of uncomplicated malaria is still a valuable preventive measure for malaria epidemics. The key point is to cure enough infections so that the remaining parasites cannot rapidly expand during seasonal transmission. Therefore, the choice of first-line malaria chemotherapy is crucial to cure those treated, as well as allow the maximum number of persons to be treated. Epidemic-prone areas of marginal transmission are good places to examine the public health consequences of treatment options because a high proportion of infections progress to symptomatic malaria. When multidrug-resistant malaria was encountered on the Thailand–Burma border, the impending epidemic was aborted by use of artemisinin combination therapy (ACT), which cured patients and blocked transmission by killing gametocytes (*30*). The most promising African example of ACT is from KwaZulu-Natal, where malaria transmission on the South Africa–Mozambique border was substantially blocked by sequentially replacing chloroquine with SP and then with ACT supplemented with residual insecticide spraying (*41*). Effective antimalarial drugs are not just good for sick African children, they can also help decrease transmission pressure in areas of seasonal transmission and stop the increasingly steep spiral of drug resistance that has now left sub-Saharan Africa without readily available treatment for uncomplicated malaria. Effective drug combinations are urgently needed not only to prevent African children from dying of malaria, but also to prevent highland malaria epidemics.
